# Dynamic Action Potential Restitution Contributes to Mechanical Restitution in Right Ventricular Myocytes From Pulmonary Hypertensive Rats

**DOI:** 10.3389/fphys.2018.00205

**Published:** 2018-03-12

**Authors:** Matthew E. L. Hardy, Eleftheria Pervolaraki, Olivier Bernus, Ed White

**Affiliations:** ^1^Multidisciplinary Cardiovascular Research Centre, University of Leeds, Leeds, United Kingdom; ^2^IHU Liryc, L'institut de Rythmologie et Modélisation Cardiaque, Fondation Bordeaux Université, Bordeaux, France; ^3^Centre de Recherche Cardio-Thoracique de Bordeaux, Université de Bordeaux, U1045, Bordeaux, France; ^4^Centre de Recherche Cardio-Thoracique de Bordeaux, Institut National de la Santé et de la Recherche Médicale, U1045, Bordeaux, France

**Keywords:** pulmonary artery hypertension, APD restitution, mechanical restitution, right heart failure, action potential clamp

## Abstract

We investigated the steepened dynamic action potential duration (APD) restitution of rats with pulmonary artery hypertension (PAH) and right ventricular (RV) failure and tested whether the observed APD restitution properties were responsible for negative mechanical restitution in these myocytes. PAH and RV failure were provoked in male Wistar rats by a single injection of monocrotaline (MCT) and compared with saline-injected animals (CON). Action potentials were recorded from isolated RV myocytes at stimulation frequencies between 1 and 9 Hz. Action potential waveforms recorded at 1 Hz were used as voltage clamp profiles (action potential clamp) at stimulation frequencies between 1 and 7 Hz to evoke rate-dependent currents. Voltage clamp profiles mimicking typical CON and MCT APD restitution were applied and cell shortening simultaneously monitored. Compared with CON myocytes, MCT myocytes were hypertrophied; had less polarized diastolic membrane potentials; had action potentials that were triggered by decreased positive current density and shortened by decreased negative current density; APD was longer and APD restitution steeper. APD90 restitution was unchanged by exposure to the late Na^+^-channel blocker (5 μM) ranolazine or the intracellular Ca^2+^ buffer BAPTA. Under AP clamp, stimulation frequency-dependent inward currents were smaller in MCT myocytes and were abolished by BAPTA. In MCT myocytes, increasing stimulation frequency decreased contraction amplitude when depolarization duration was shortened, to mimic APD restitution, but not when depolarization duration was maintained. We present new evidence that the membrane potential of PAH myocytes is less stable than normal myocytes, being more easily perturbed by external currents. These observations can explain increased susceptibility to arrhythmias. We also present novel evidence that negative APD restitution is at least in part responsible for the negative mechanical restitution in PAH myocytes. Thus, our study links electrical restitution remodeling to a defining mechanical characteristic of heart failure, the reduced ability to respond to an increase in demand.

## Introduction

Action potential duration (APD) restitution describes the relationship between APD and the preceding diastolic interval. Typically as diastolic interval decreases the APD at late repolarization also decreases (Carmeliet, 2004; Weiss et al., 2005; Taggart and Lab, 2008). Dynamic APD restitution is thought to be dependent upon the stimulation frequency-dependent changes in activation, inactivation, and electrochemical driving force of the ion channels and electrogenic exchangers that underlie the action potential. As such it is likely to be dependent upon cardiac region and species (Sallé et al., 2008; O'Hara et al., 2011) due to variations in the relative expression of ion channels and exchangers. Various mechanisms are thought to influence APD restitution, with evidence for the involvement of Kv channels, (Shimoni et al., 1994; Rocchetti et al., 2006); L-type Ca^2+^ current (I_CaL_) (Li et al., 1999; Fauconnier et al., 2003) and sodium-calcium exchange (NCX) (Nanasi et al., 1996; Janvier et al., 1997). The rate-dependent properties of the late Na^+^ current have more recently been associated with APD restitution characteristics (Morita et al., 2011) and this current has been reported to be upregulated in the monocrotaline (MCT) rat model of RV failure (Rocchetti et al., 2014) used in this study. Steepening of APD restitution is seen in many pathological conditions and is thought to be pro-arrhythmic (Selvaraj et al., 2007; Keldermann et al., 2008).

Contractile activity is also subject to restitution. In the normal rat the relationship has been described as positive, negative or biphasic depending on experimental conditions. Variability in response was linked to stimulation frequency-dependent changes in [Na^+^]i (via NCX activity) and sarcoplasmic reticulum (SR) Ca^2+^ load (Frampton et al., 1991). The inactivation of I_CaL_ is also thought to be an important factor in determining SR Ca^2+^ load and diastolic [Ca^2+^]i in balancing Ca^2+^ influx and efflux (Dibb et al., 2007). Dysfunctional Ca^2+^ handling is seen in heart failure (Houser et al., 2000) and the relative importance of a given mechanism can vary with the exact conditions of failure. However, negative mechanical restitution, whereby contractility declines as stimulation frequency is increased, is a manifestation of an inability to respond to increased demand, and is a defining characteristic of heart failure.

Pulmonary artery hypertension (PAH) causes the right ventricle (RV) to become hypertrophied and eventually to fail. During this process the RV undergoes electrophysiological remodeling where prolonged repolarization is linked with increased mortality (Rich et al., 2013). Similar electrical remodeling, including steeper APD restitution, is seen in the MCT rat model of PAH and RV failure (Piao et al., 2010; Benoist et al., 2011, 2012). This model is also characterized by a steep negative contraction-frequency relationship (Benoist et al., 2012; Fowler et al., 2015, 2018; Natali et al., 2015). This mechanical response is linked to a more steeply decreasing [Ca^2+^]i transient amplitude (Benoist et al., 2012). The contractile response could be the result of dysfunction of Ca^2+^ handling mechanisms, for example there is decreased expression and function of the sarcoplasmic reticulum Ca^2+^ uptake pump (SERCA) in the MCT model (Piao et al., 2010; Benoist et al., 2012; Fowler et al., 2015).

However another, to date untested, possibility is that contraction in the MCT model is influenced by the steep negative APD restitution via mechanisms that link APD and contraction. AP profile and [Ca^2+^]i transient profile are interlinked. The shape of the AP can influence Ca^2+^ homeostasis by modulation of processes such as the inactivation of I_CaL_ and the extrusion of Ca^2+^ by NCX (Bouchard et al., 1995; Clark et al., 1996). Modulation of these Ca^2+^ entry and extrusion pathways influences SR Ca^2+^ load and subsequent SR Ca^2+^ release. Conversely, changes in Ca^2+^ homeostasis will affect electrogenic Ca^2+^ dependent processes and thereby modulate AP profile (Mitchell et al., 1984a,b). If changes in the AP affect the [Ca^2+^]i transient, contraction will be affected.

In this study we wished to further investigate the causes of steeper APD restitution in the RV myocytes of PAH rats. Additionally, we wished to test the hypothesis that negative APD restitution causes negative mechanical restitution in these myocytes. In order to independently control stimulation frequency and membrane depolarization profile, we used voltage clamp techniques to mimic APD restitution in RV myocytes from control and PAH animals, whilst simultaneously measuring cell shortening.

## Materials and methods

### Animal model

Experiments were conducted with local ethical approval and in accordance with UK Home Office and European Parliament Directive 2010/63/EU guidelines on the use of animals in research. Male Wistar rats (200 g) were given a single i.p. injection of monocrotaline (60 mg/kg) to induce PAH and RV failure (MCT) or an equivalent volume of saline (controls, CON). External signs of heart failure became apparent in MCT animals 26 ± 1 days after injection. Heart failure signs (weight loss on consecutive days, lethargy, piloerection, cold extremities) are well linked with cardiovascular dysfunction in this model (e.g., Hardziyenka et al., 2006). Upon presentation of heart failure signs, MCT animals were killed and hearts excised, CON animals were killed on time matched days.

### Electrophysiology and myocyte shortening measurements

Single RV myocytes were isolated by perfusion of the extracted heart with collagenase as previously described (Benoist et al., 2012; Fowler et al., 2015). Isolated myocytes were placed in the experimental chamber of a Nikon Diphot inverted microscope and superfused with a solution that contained in (mM): NaCl 135; KCl 6, NaH_2_PO_4_ 0.33, HEPES 10, Glucose 5.6, Na Pyruvate 5, CaCl_2_ 2, MgCl_2_ 1, pH 7.4. Action potentials and ionic currents were recorded in whole cell configuration using pipettes with a resistance of 3–6 MΩ filled with a solution containing (in mM): KCl 130; MgCl_2_ 5; Na_2_ATP 5; HEPES 10 at pH 7.15. Junction potential (1 mV) and series resistance were compensated. An Axoclamp 2B amplifier was used in bridge mode to record action potentials and in discontinuous voltage clamp mode, at a switching frequency of 4 KHz, to voltage clamp myocytes. In studies investigating ranolazine, sharp microelectrodes filled with 0.6 M KCL with a resistance of 20–30 mOhms were used. To buffer intracellular Ca^2+^ transients some cells were incubated for 15 min with 5 μM BAPTA-AM at room temperature. To investigate late Na^+^ current, action potentials were recorded from myocytes before and after 5 min. perfusion with 5 μM ranolazine. All experiments were performed at 37°C.

Dynamic restitution characteristics were measured. Action potentials were stimulated by current injection at 25% above threshold level at stimulation frequencies between 1 and 9 Hz. APD was measured at 25% (APD25) and 90% (APD90) of repolarization. In some experiments the effect of imposed current on APD was investigated by injection of negative current 50 to 500 pA in amplitude for 150 ms beginning 5 ms after the upstroke of the action potential.

In action potential clamp (AP clamp) experiments, action potentials were recorded at 1 Hz stimulation frequency and this waveform used to voltage clamp that cell at stimulation frequencies between 1 and 7 Hz.

In experiments combining voltage clamp and the measurement of cell shortening, cells were voltage clamped with an AP-like waveforms that mimicked those of CON and MCT myocytes recorded at 1 and 5 Hz stimulation frequency and which reflected the APD restitution characteristics of these cells. CON myocytes were depolarized for 50 ms at both 1 and 5 Hz while MCT myocytes were depolarized for 125 ms at 1 Hz and 50 ms at 5 Hz (see Benoist et al., 2012). Electrophysiological data was acquired at 2 KHz and analyzed with pClamp (Axon Instruments). Ionic currents were normalized to cell capacitance. Cell shortening was measured by video edge detection (VED-114, Crystal Biotech) from the video image of the cell. Shortening data was sampled at 200 Hz and simultaneously recorded with electrical activity in pClamp. Shortening parameters were measured on the averaged trace of 4–8 contractions at steady state. The amplitude of shortening and the time from peak shortening to 50% relaxation were calculated.

### Statistical analysis

Data are expressed as mean ± sem. Statistical comparisons of two-sample CON and MCT data were made by unpaired *t*-test. Two-way repeated measures ANOVA with *post-hoc* Sidak pairwise comparisons were used for restitution and AP clamp data. Paired *t*-tests were used to compare the effect of 1 vs. 5 Hz frequency stimulation on myocyte shortening.

## Results

**Figure 1:**
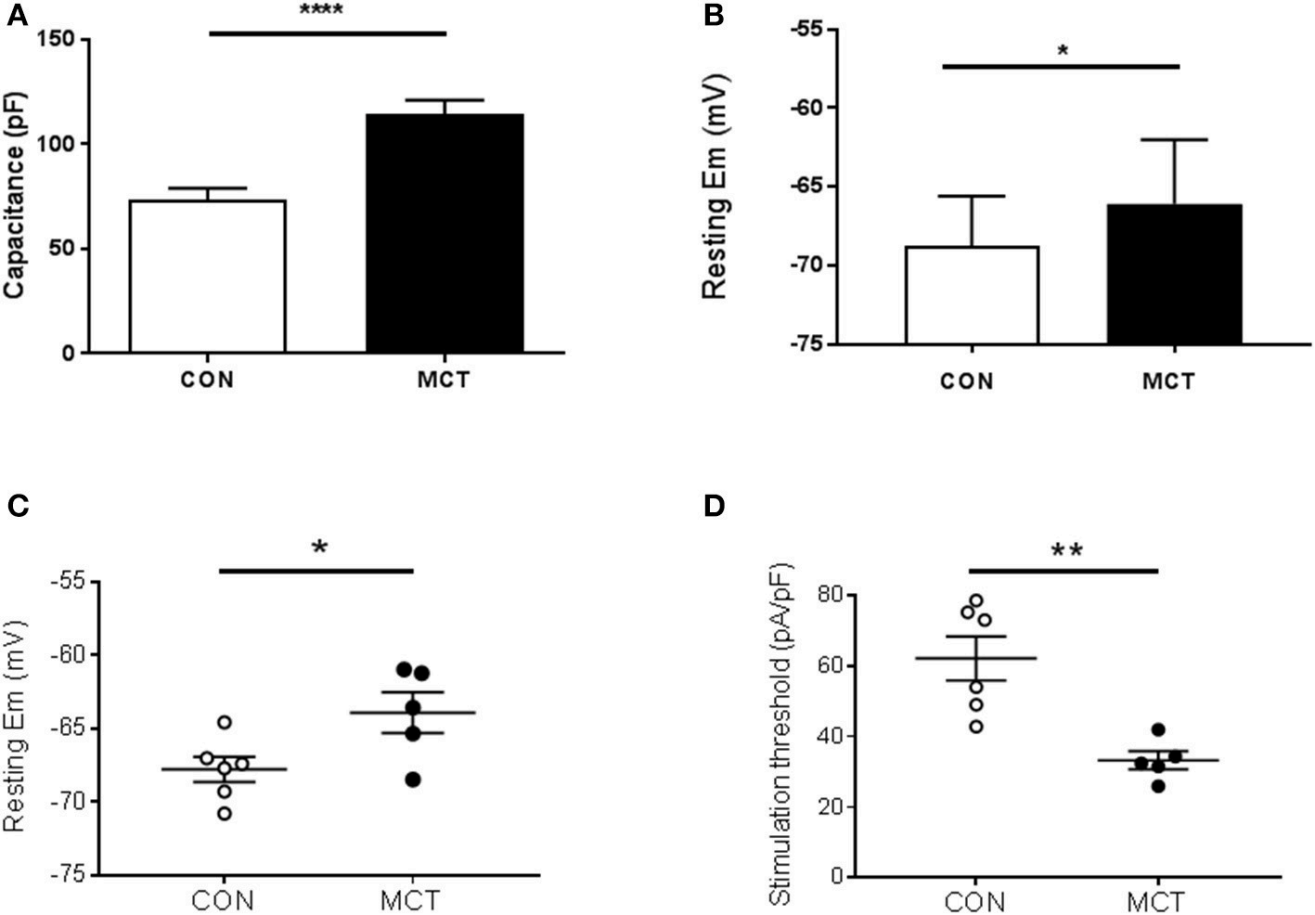
**(A)** RV myocyte capacitance was greater in MCT myocytes than CON myocytes, consistent with hypertrophy in PAH. **(B)** Resting membrane potential was more depolarized in MCT than CON myocytes. Resting membrane potential **(C)** and current density required to trigger an action potential **(D)** measured in the same cells, were lower in MCT than CON myocytes. **(A)** *n* = 15 CON, 20 MCT myocytes, **(B)**, 20 CON, 21 MCT **(C,D)** *n* = 6 CON, 5 MCT myocytes, ^****^P < 0.0001, ^**^P < 0.01, ^*^P < 0.05 unpaired *t*-test.

**Figure 2:**
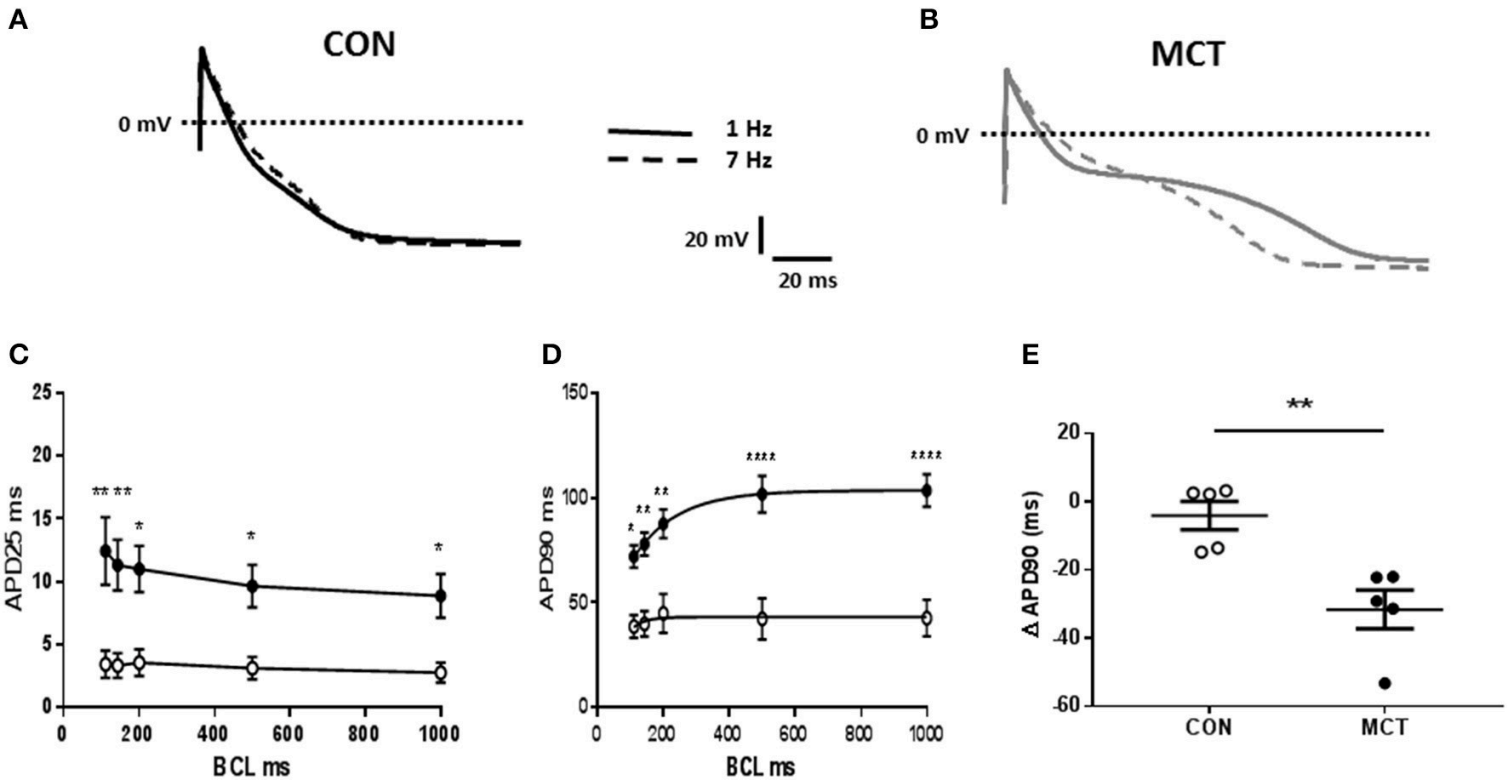
Representative APs from **(A)** CON and **(B)** MCT RV myocytes at stimulation frequencies of 1 and 7 Hz (dashed lines) **(C)** APD25 restitution curves **(D)** APD90 restitution curves for CON (°) and MCT (•) myocytes **(E)** change in APD90 from stimulation frequencies of 1–9Hz. Increasing stimulation frequency caused a larger fall in APD90 in MCT than CON myocytes. *n* = 5 CON and 5 MCT myocytes ^****^P < 0.0001, ^**^P < 0.01, ^*^P < 0.05 CON vs. MCT **(C,D)** two way RM ANOVA, **(E)** unpaired *t*-test.

When MCT animals showed signs of heart failure there was increased heart weight:body weight, indicating cardiac hypertrophy (*P* < 0.01 Table 1). Consistent with this observation the capacitance of MCT RV myocytes was larger than CON myocytes (*P* < 0.0001, Figure 1A). The diastolic membrane potential of MCT myocytes was less polarized than CON myocytes (*P* < 0.05 Figures 1B,C) and the current density needed to trigger an action potential was less in MCT myocytes (*P* < 0.01, Figure 1D). Between stimulation frequencies of 1–9Hz, APD25 and APD90 was longer in MCT myocytes (Figures 2A–D) and the change in APD90 was greater (*P* < 0.01, Figures 2C–E).

**Table 1:** Whole body and organ weights for CON and MCT animals.

	**CON**	**MCT**
Animals (*n*)	6	6
Body weight (g)	359 ± 5.7	275 ± 8^***^
Heart Weight (g)	1.48 ± 0.06	1.63 ± 0.09
Lung Weight (g)	1.82 ± 0.03	3.26 ± 0.31^***^
HW:BW (mg/g)	4.13 ± 0.17	5.95 ± 0.38^**^
LungW:BW (mg/g)	5.07 ± 0.06	12.03 ± 1.40^***^

*HW, heart weight; BW, body weight. MCT animals displayed cardiac hypertrophy and enlarged lungs, consistent with PAH*.

*** *P < 0.001*,

** *P < 0.01*,

* *P < 0.05, unpaired t-test*.

**Figure 3:**
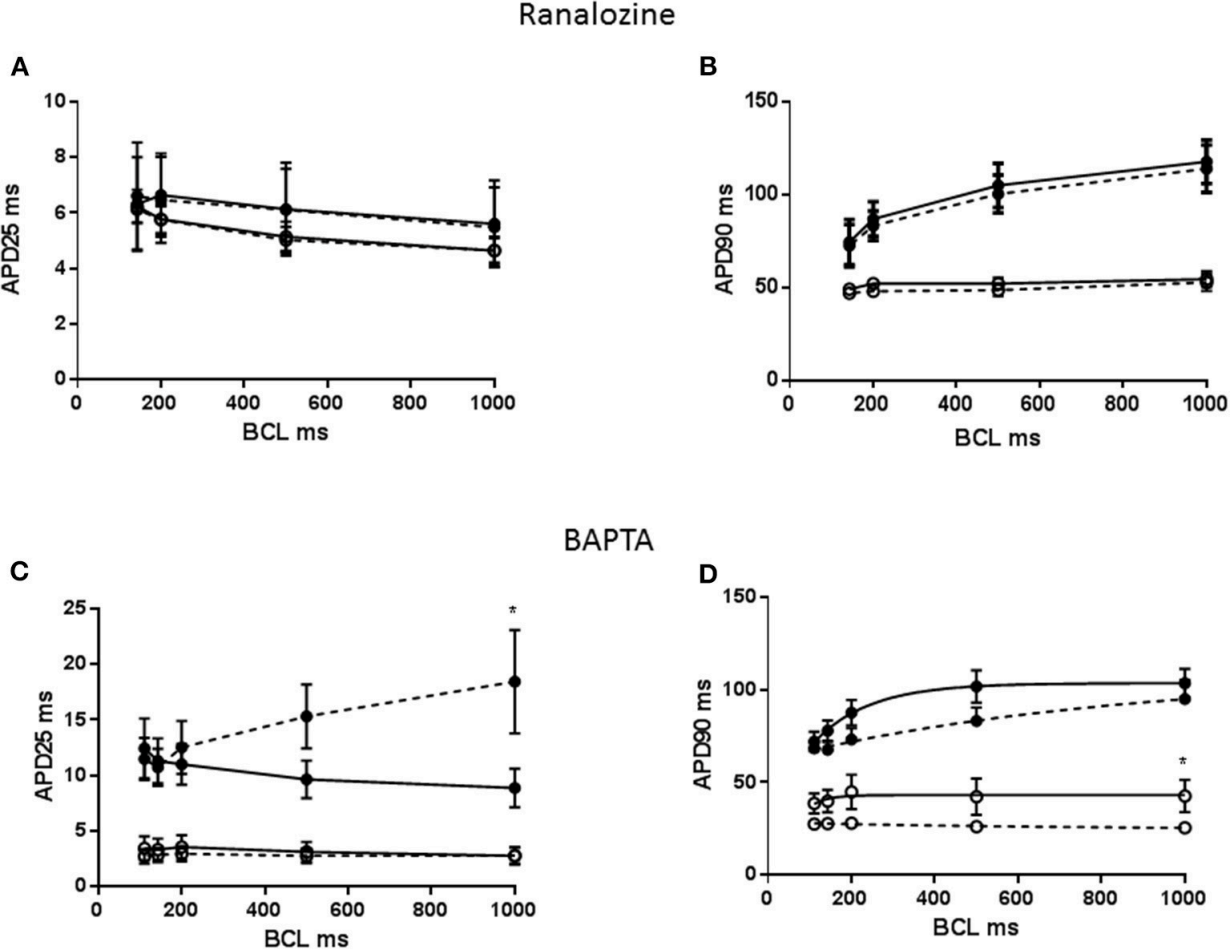
**(A)** APD25 restitution and **(B)** APD90 restitution for CON (°) and MCT (•) RV myoctes in the presence (dashed lines) or absence of 5 μM ranolazine. Ranolazine had no effect on APD25 or APD90. **(C)** APD25 restitution and **(D)** APD90 restitution for CON (°) and MCT (•) RV myocytes in the presence (dashed lines) or absence of prior exposure to BAPTA. BAPTA lengthened APD25 and reversed the restitution curve in MCT myocytes. In CON myocytes BAPTA reduced APD90 but did not affect APD restitution. *n* = 6–9 myocytes **(A,B)**, *n* = 5–6 **(C,D)**. ^*^P < 0.05 vs. ranolazine or BAPTA, two way RM ANOVA.

APD restitution has been linked to late Na^+^ channel, however APD restitution was not modified in either MCT or CON myocytes by prior exposure to 5 μM ranolazine (*P* > 0.05, Figures 3A,B). To test the influence of [Ca^2+^]i transients on APD restitution, myocytes were pre-exposed to the Ca^2+^ buffer, BAPTA-AM. APD25 lengthened in FAIL myocytes at 1 Hz (*P* < 0.05) and the direction of the APD25 restitution was reversed (Figure 3C) APD90 was shortened in CON myocytes at 1 Hz (*P* < 0.05, Figure 3D) but there was little effect on the shape on the APD90 restitution relationship.

**Figure 4:**
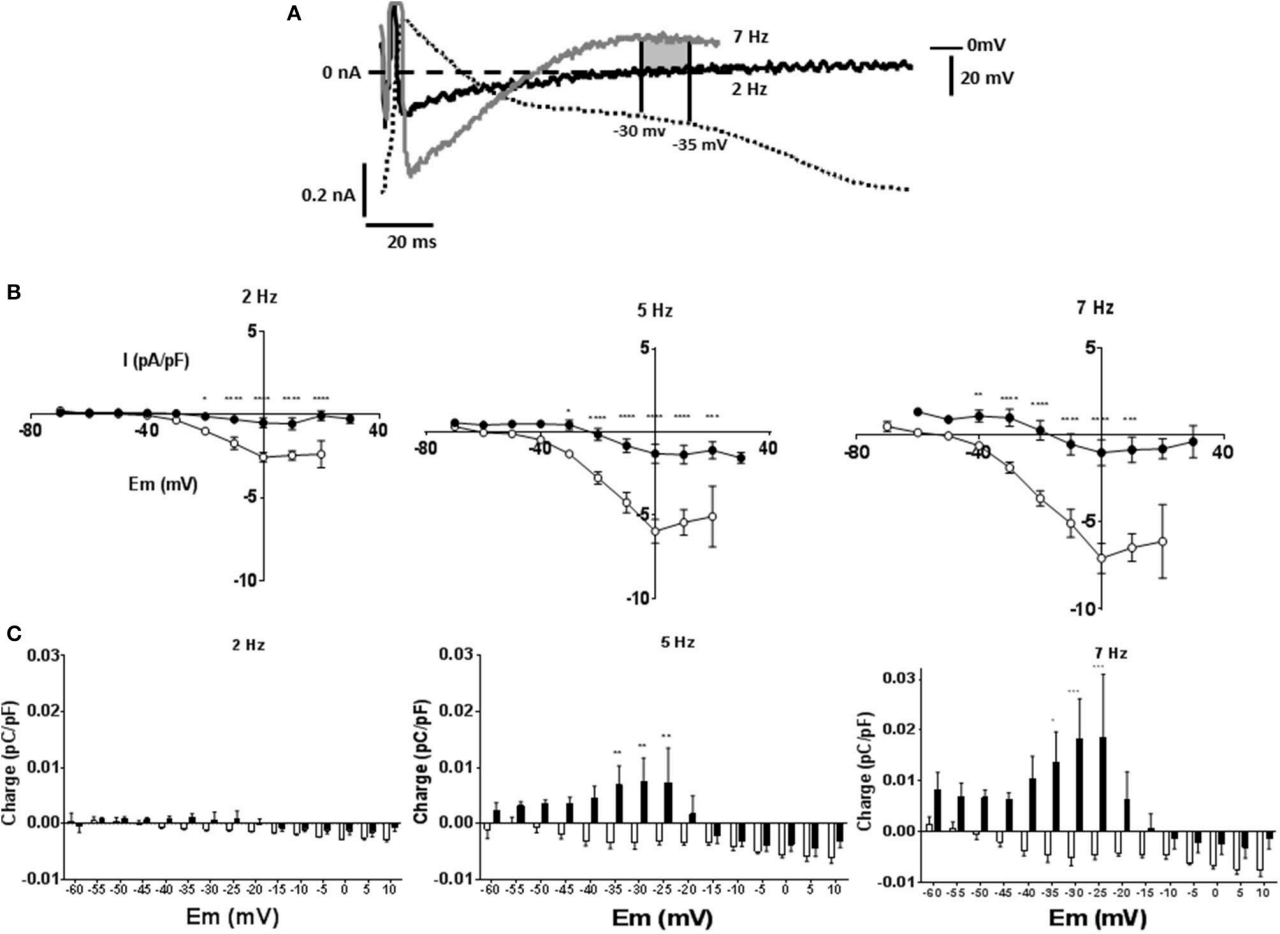
Myocytes were voltage clamped with the waveform of their own action potential recorded at 1 Hz (AP clamp). Stimulation frequency was increased and currents recorded under AP clamp. **(A)** Experimental traces illustrating the measurement of membrane current and charge described in panels **(B,C)** and in Figure 5. A cell is voltage clamped with its free action potential profile recorded at 1 Hz. Current traces are shown when stimulation frequency is increased to 2 and 7 Hz (subtracted from any current recorded at 1 Hz). As stimulation frequency increased an inward current developed early in repolarization and an outward current latter in repolarization. The charge was calculated as the area of current ^*^ time passed during a 5 mV change in voltage. The shaded area shows charge passed between the membrane potentials of −30 and −35 mV at a stimulation frequency of 7 Hz, there was minimal current or charge at 2 Hz within this voltage range. **(B)** Current voltage relationships for 1 Hz subtracted currents at 2, 5, and 7 Hz in CON (°) and MCT (•) myocytes. Inward current early in the simulated AP repolarization was larger in CON than MCT. **(C)** Charge measured at 5 mV sampling intervals during AP clamp. In CON myocytes charge was predominantly inward whilst in MCT the largest charge was outward. *n* = 7 CON and 7 MCT myocytes ^****^P < 0.0001, ^***^P < 0.001, ^**^P < 0.01, ^*^P < 0.05 CON vs. MCT two way RM ANOVA.

**Figure 5:**
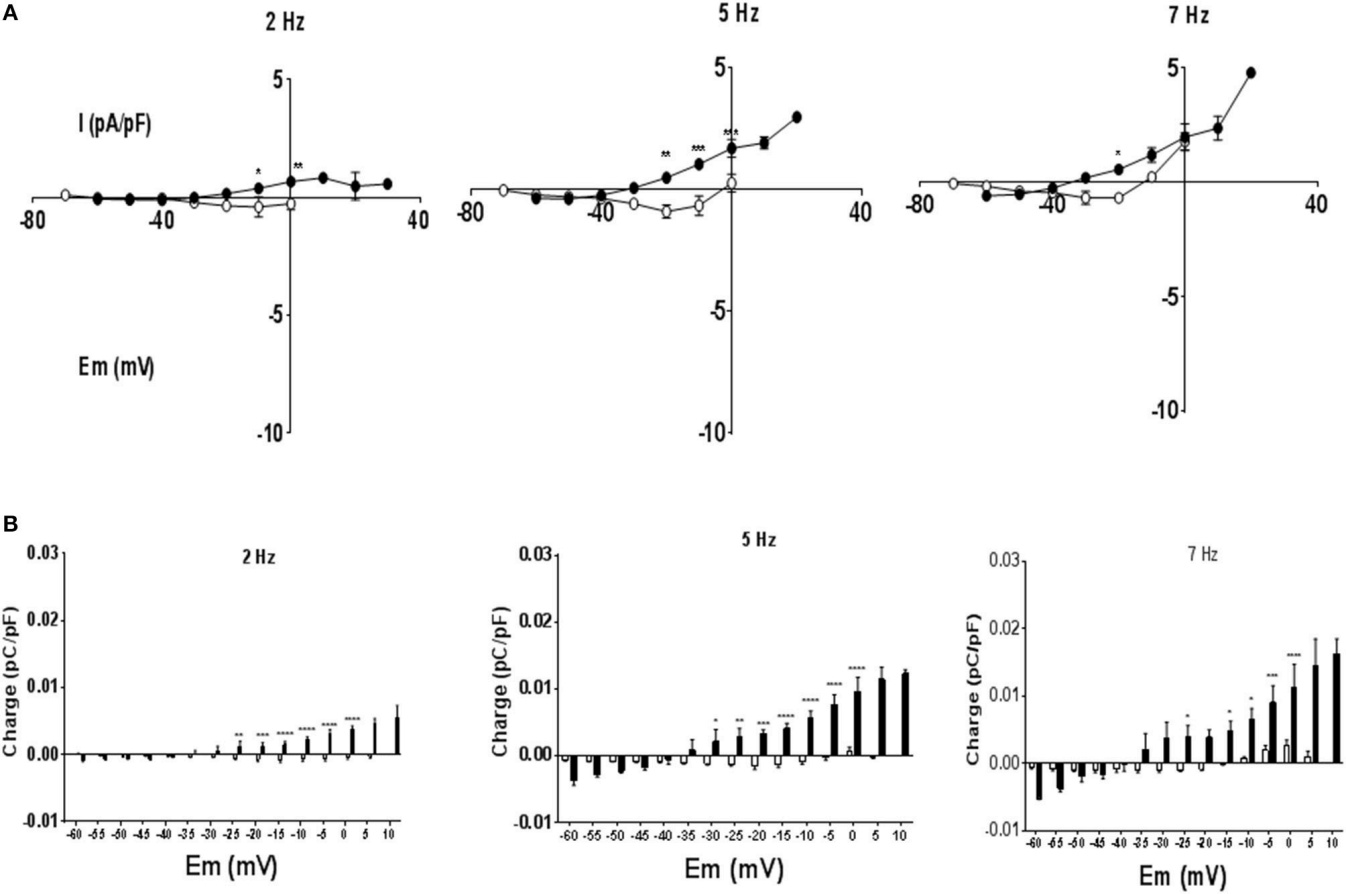
The experimental protocol described in Figure 4 was repeated following exposure to BAPTA. **(A)** BAPTA abolished inward currents at early repolarization potentials (see Figure 4) to reveal a net outward current at these potentials. **(B)** There was little charge movement in CON cells but outward charge at positive membrane potentials in MCT myocytes. *n* = 4 CON and 3 MCT myocytes exposed to BAPTA ^****^P < 0.0001, ^***^P < 0.001, ^**^P < 0.01, ^*^P < 0.05 CON vs. MCT two way RM ANOVA.

To investigate currents underlying rate dependent changes in APD, myocytes were voltage clamped with their own AP waveform recorded at 1 Hz (AP clamp). Under AP clamp the current recorded at 1 Hz stimulation frequency was close to zero, however as stimulation frequency was increased to 7 Hz the current evoked increased. Figure 4A shows currents at 2 and 7 Hz for a MCT myocyte, after subtraction of any current recorded at 1 Hz. At 7 Hz there was an initial inward current that shifted to an outward current as the AP clamp mimicked late AP repolarization. Current voltage relationships are given in Figure 4B. Although the rate dependent change in free APD was greater in MCT myocytes (see Figure 2) currents recorded under AP clamp were smaller in MCT myocytes (Figure 4B). Charge (measured in 5 mV sections, see shaded area Figure 4A), was greater in MCT (Figure 4C) despite reduced current amplitude due to the longer AP clamp pulse. In the presence of BAPTA the inward currents (Figure 5A) and inward charge (Figure 5B) were abolished and were replaced by net outward current and charge.

**Figure 6:**
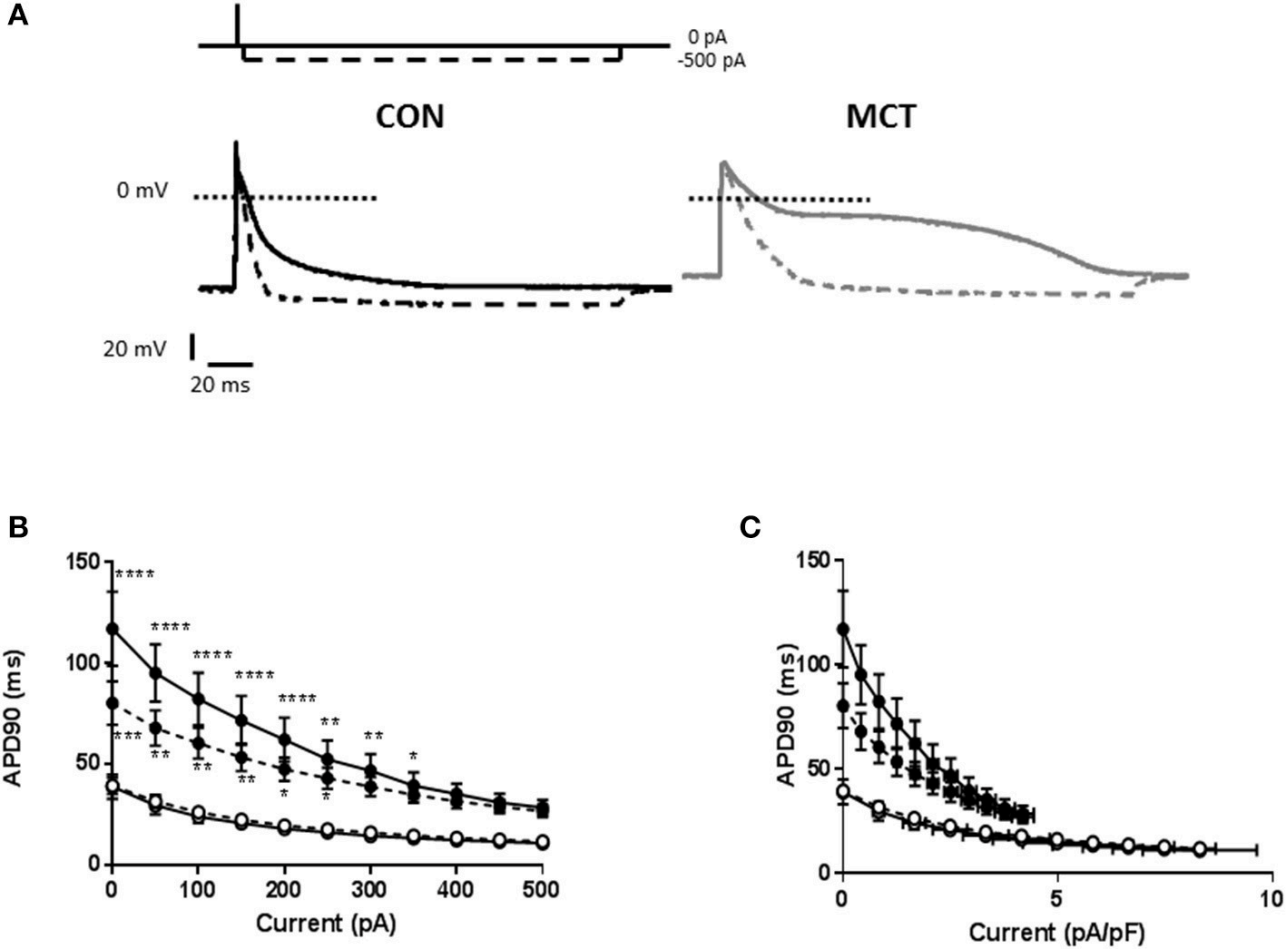
**(A)** Representative APs from a CON and MCT myocyte. When a 150 ms, −500 pA hyperpolarizing pulse was applied shortly after the AP upstroke, the APD was shortened (dashed line). The shortening was greater in the MCT myocyte. **(B)** APD90 in CON (°) and MCT (•) myocytes in the presence of hyperpolarizing pulses from 0 to −500 pA at 1 and 5 Hz (dashed lines). The shortening effect of a given current amplitude was greater in MCT myocytes than CON. Increasing stimulation frequency attenuated the shortening in MCT myocytes but had no effect in CON myocytes. **(C)** A similar profile of APD shortening to that described in **(B)** was observed when hyperpolarizing current was normalized to cell capacitance. *n* = 6 CON and 7 MCT myocytes ^****^P < 0.0001, ^***^P < 0.001, ^**^P < 0.01, ^*^P < 0.05 CON vs. MCT at the same stimulation frequency two way RM ANOVA.

To further investigate the intrinsic properties of MCT and CON myocytes, cells were stimulated at 1 and 5 Hz and 150 ms hyperpolarizing currents between 50–500 pA in amplitude were applied 5 ms after the upstroke of the action potential (Figure 6A). It was observed that a given hyperpolarizing current had a greater shortening effect upon MCT myocytes than CON, whether measured as absolute current (Figure 6B) or current normalized to cell capacitance (Figure 6C). Increasing stimulation frequency did not modulate the effect of hyperpolarizing current in CON myocytes but reduced its effect in MCT (Figures 6B,C).

**Figure 7:**
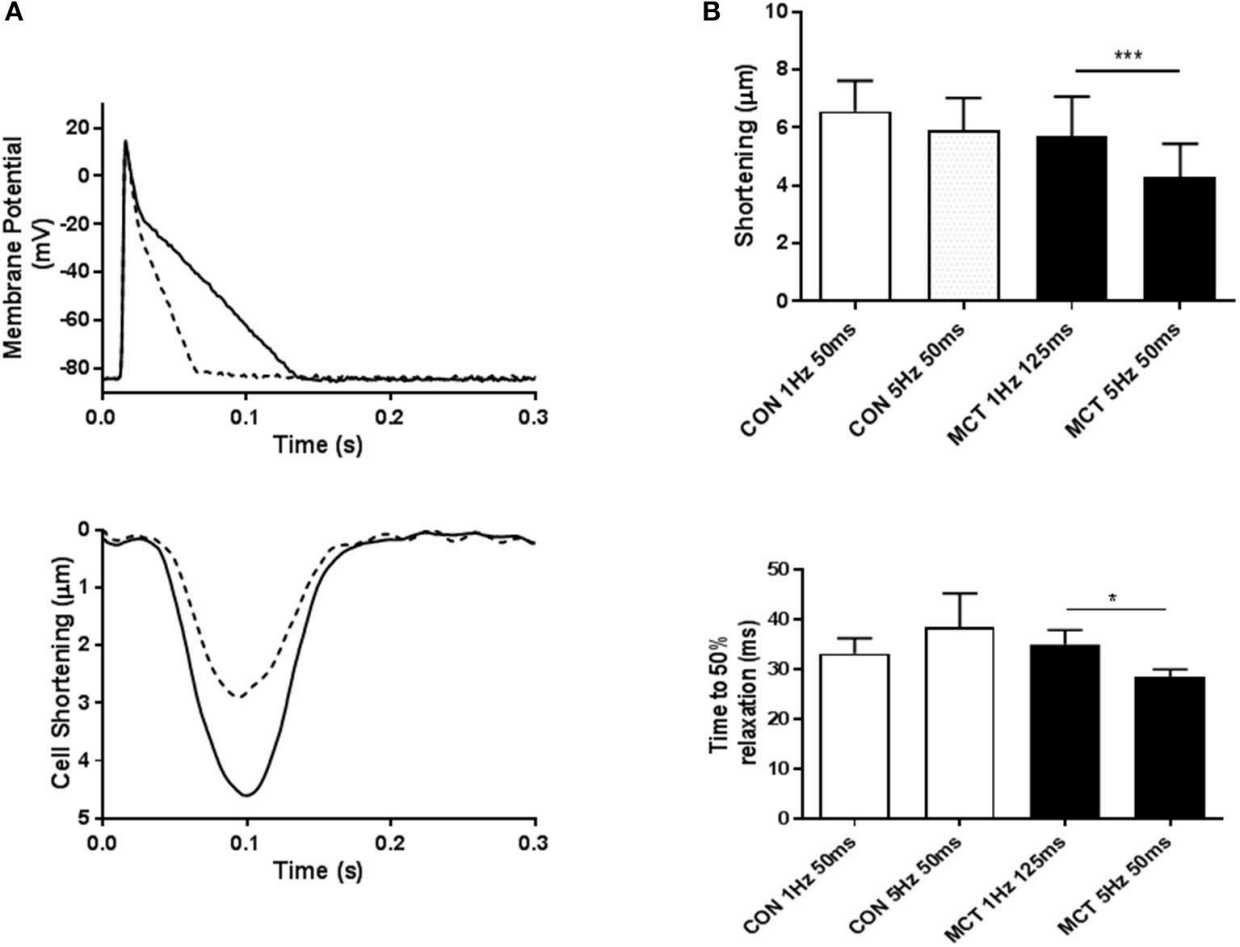
**(A)** When the voltage clamp profile was changed from mimicking a long to a short (dashed line) AP, the associated cell shortening (dashed line) decreased, demonstrating that AP profile can influence contraction. **(B)** Cell shortening and time to 50% relaxation in response to voltage clamp protocols mimicking APD restitution. For CON myocytes depolarization duration was 50 ms at both 1 and 5 Hz, for MCT myocytes depolarization was 125 ms at 1 Hz and 50 ms at 5 Hz. Increasing stimulation frequency had no effect on the amplitude or time course of contraction in CON myocytes but reduced both in MCT myocytes. Thus, mimicking APD restitution in MCT myocytes provoked mechanical restitution. *n* = 11 CON and 11 MCT myocytes. ^*^P < 0.05, ^***^P < 0.001, paired *t*-test 1 vs. 5 Hz for CON and MCT.

**Figure 8:**
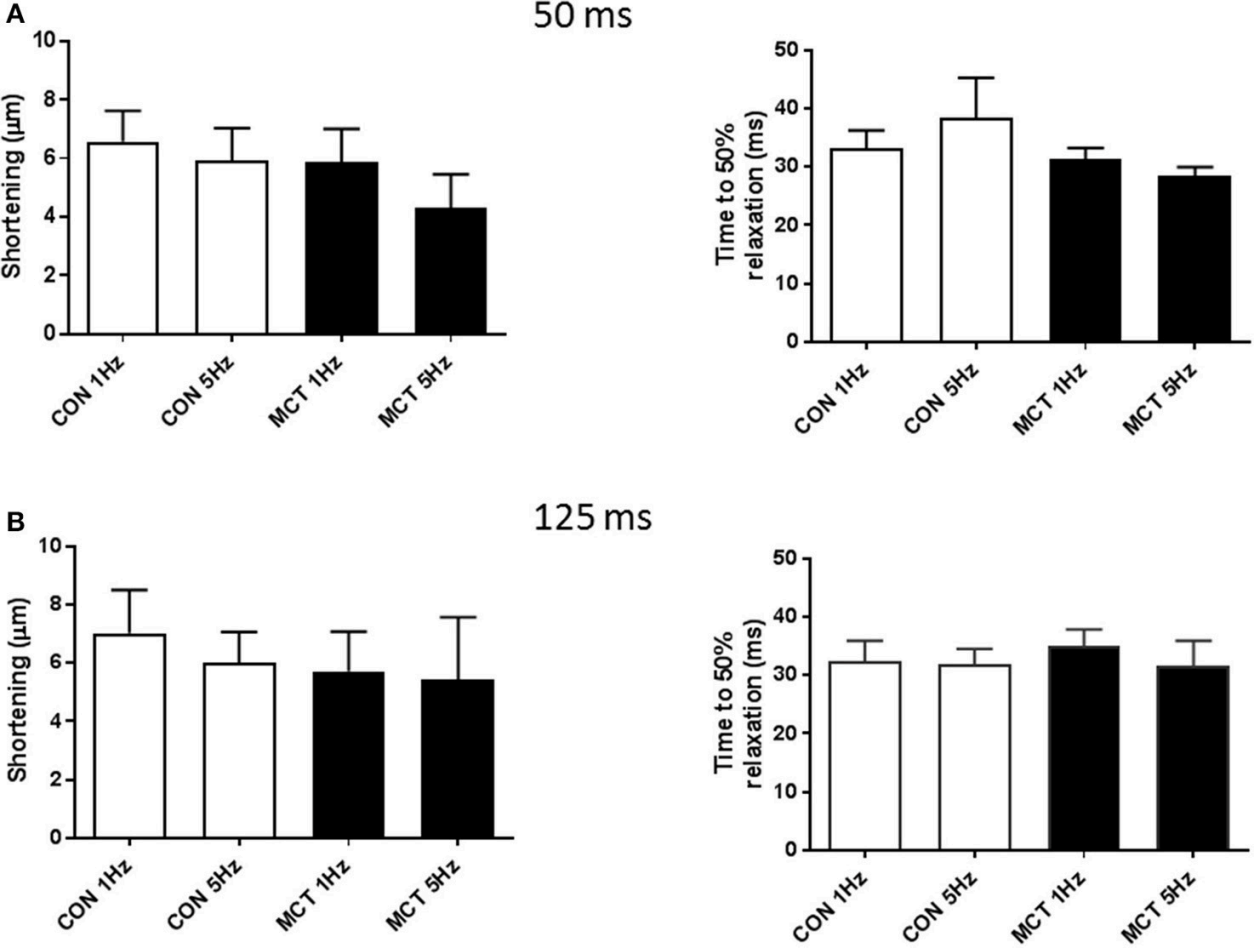
Cell shortening and time to 50% relaxation at 1 and 5 Hz when voltage clamp depolarization was maintained at either **(A)** 50 ms or **(B)** 125 ms. When depolarization duration was maintained there were no changes in contraction amplitude or relaxation in either group. Thus, in MCT myocytes when APD restitution was absent, mechanical restitution was absent. *n* = 11 CON and 11 MCT myocytes, paired *t*-tests 1 vs. 5 Hz for CON and MCT.

Figure 7A demonstrates that APD profile can modify cell shortening, at a fixed stimulation rate of 1 Hz, shortening the voltage clamp pulse from 125 to 50 ms led to a decrease in myocyte shortening. Voltage clamp protocols were used to mimic the APD90 restitution of CON and MCT myocytes. An increase in stimulation frequency from 1 to 5 Hz did not affect the amplitude or time course of contraction in CON myocytes (*P* > 0.05 Figure 7B). In contrast increasing frequency and decreasing depolarising pulse duration in MCT myocytes reduced both shortening amplitude (*P* < 0.001) and relaxation time (*P* < 0.05) (Figure 7B). However, when stimulation frequency was increased but depolarization duration was held constant, at either 50 ms (Figure 8A) or 125 ms (Figure 8B) contractile parameters were not changed in either group of cells (*P* > 0.05).

## Discussion

Our observations that RV myocytes from MCT-treated animals are hypertrophied, have longer APD and steeper APD restitution when compared with myocytes from saline-treated animals agree with previous observations from this model (e.g., Benoist et al., 2012). Our study provides new information on APD restitution and membrane potential stability that have implications for arrhythmias and we propose a likely cause of these effects. We also present novel information on the role that steep negative APD restitution can have on contractile function in heart failure with respect to the decreased ability of failing myocardium to respond to an increase in demand.

### Effects of ranolazine and BAPTA on APD restitution

Electrical restitution is important in the development of cardiac arrhythmias, in particular ventricular fibrillation, due to its role in the fragmentation of activation wave fronts (Weiss et al., 2005). Recent research has proposed a role for late Na^+^ current in the generation of arrhythmias, based on the anti-arrhythmic effect of ranolazine and its effect on APD restitution (Morita et al., 2011), however we observed no effects of ranolazine on APD restitution in rats.

APD restitution is likely to be the result of changes in multiple ion channels and electrogenic exchangers which vary with species and experimental conditions (Janvier et al., 1997; O'Hara et al., 2011). The buffering of [Ca^2+^]i by BAPTA may delay Ca^2+^ dependent inactivation of L-type current and explain the prolonged APD25 seen in MCT myocytes at 1 Hz. That this effect was not seen in CON myocytes and reduced with increasing stimulation frequency in MCT myocytes is evidence that the long MCT APD recorded at 1Hz is more easily manipulated by interventions (see below). The later phase of repolarization of the rat AP is thought to be supported by NCX (e.g., Mitchell et al., 1984b; Janvier et al., 1997), the application of BAPTA reduces forward mode NCX and shortens APD90.

### AP clamp experiments

In theory, zero current is evoked when a cell is AP clamped with its own AP waveform, unless some intervention is applied (Doerr et al., 1990), therefore our experiments do not address why the AP profile at 1 Hz is altered by MCT or by BAPTA. However, currents evoked by increasing stimulation frequency above 1 Hz (Figures 3, 4) can be interpreted in the context of stimulation frequency dependent changes in APD (Figures 2, 3). In general, current amplitudes were larger in CON myocytes, the greater charge in MCT is explained by longer depolarization durations.

Net current in the CON IV relationship was predominantly inward and associated with the depolarized membrane potentials of the early AP. Exposure to BAPTA abolished these inward currents, suggesting they are carried by forward mode NCX, and revealed outward currents. It was suggested (Nanasi et al., 1996; Janvier et al., 1997) that NCX plays an important role in rat APD restitution. However, whether the currents were inward or outward, the effect on APD was small, suggesting the normal rat APD is relatively resistant to modulation.

In MCT myocytes, the development of inward currents at more depolarized voltages is consistent with the lengthening of APD25 but the major charge movement was outward, occurring at negative membrane potentials and consistent with the stimulation frequency-dependent shortening of the APD90. In the absence of an effect of ranolazine and little effect of BAPTA on the MCT APD restitution, candidates are inactivation of I_CaL_ and increased steady state activation of K^+^ currents, both would be recorded as more outward current under AP clamp. However, we have previously shown that the stimulation frequency-dependent change in both the amplitude and inactivation of I_CaL_, using conventional square pulse voltage clamp, are not different between MCT and CON myocytes (Benoist et al., 2012). In addition, the IV relationship of the stimulation frequency dependent current of MCT myocytes in Figure 4 is more suggestive of K^+^ currents than Ca^2+^-currents.

### Interpretation of current injection and implications for membrane stability and arrhythmias

RV MCT myocytes required less current to trigger an action potential (Figure 1D) or to shorten APD (Figure 6). Furthermore, despite the greater stimulation frequency-dependent change in APD in MCT myocytes, currents evoked under AP clamp were smaller in MCT than CON. These observations demonstrate that the resting membrane potential and AP profile of MCT myocytes are less stable and more easily perturbed than CON myocytes. If less current is required to modulate resting membrane potential and AP profile, delayed after depolarization and early after depolarization arrhythmias will be easier to generate. Indeed, it has been shown that the MCT hearts are pro-arrhythmic (Benoist et al., 2011, 2012; Umar et al., 2012). These observations are important because many of the changes in electrophysiology seen in MCT rats are also seen in human PAH patients (e.g., Rich et al., 2013).

RV MCT myocytes express reduced levels of multiple Kv channels and this is thought to explain the longer APD (via less I_to_ and rodent equivalent I_K_ current) and the decreased resting membrane potential (via reduced I_K1_) (Lee et al., 1997, 1999; Zhang et al., 2004; Piao et al., 2010; Benoist et al., 2011, 2012). Reduction in Kv channels may also explain the increased susceptibility to injected current, in the presence of less Kv currents, membrane resistance will be increased and the membrane potential change for any given imposed current will be greater (V=IR). This interpretation has interesting implications for other stimulation frequency-dependent currents. For example, if the fall in I_CaL_ current density is the same in MCT and CON myocytes (as we have shown, Benoist et al., 2012) this should have greater impact on the MCT APD than the CON APD.

### Influence of APD restitution on contraction

It is a characteristic of the MCT model that in FAIL myocytes contraction shows negative restitution whilst CON myocytes show a biphasic response (Benoist et al., 2012; Fowler et al., 2015, 2018; Natali et al., 2015). These contractile responses are linked to changes in the amplitude of the [Ca^2+^]i transient (Benoist et al., 2012). The inability of MCT cells to maintain contraction in response to an increase in demand fulfills a definition of heart failure. This response may arise because Ca^2+^ handling is compromised e.g., by reduced SERCA function (Piao et al., 2010; Benoist et al., 2012, 2014) or by compromised myofilament function. Both processes could be related to decreased levels of creatine kinase (Fowler et al., 2015) that maintains ATP:ADP locally in areas of high ATP turnover (Guzun et al., 2011).

But contraction can also be modulated by APD profile, a longer APD can delay L-type Ca^2+^ current inactivation and reduce Ca^2+^ extrusion via forward mode NCX thus increasing SR Ca^2+^ load and contraction amplitude (Terrar and White, 1989; Bouchard et al., 1995; Clark et al., 1996). This effect is demonstrated in Figure 7A. Therefore, contraction in MCT myocytes at low (1 Hz) stimulation frequency may be supported by the long APD but this effect is progressively lost as stimulation frequency increases and APD falls. When stimulation frequency increased, a fall in MCT myocyte contraction was only seen when depolarization duration was also reduced (mimicking APD restitution) (Figure 7B) and not when depolarization duration was fixed (Figure 8). We therefore conclude that negative APD restitution is at least in part responsible for negative contractile restitution in MCT myocytes.

### Limitations

Membrane potential, underlying currents and [Ca^2+^]i are interconnected. Alteration of one factor is likely to have multiple repercussions and this should be acknowledged when interpreting AP clamp experiments. Both electrical and mechanical activity are modulated by mechanical and electrical loading in multicellular preparations, single myocyte studies do not address these factors.

## Conclusion

In MCT myocytes, APD restitution contributes to mechanical restitution and smaller currents are needed to modify the AP profile of MCT myocytes, decreasing membrane potential stability and increasing the susceptibility to arrhythmias. Incorporating this new information with existing knowledge, we propose that electrical instability is caused by decreased expression of Kv channels and the effects on mechanical restitution are due to reduced I_CaL_ and increased Ca^2+^ extrusion by NCX. APD prolongation and steep APD restitution are common to many heart failure phenotypes, thus our observations may be relevant to other cardiac pathologies.

## Author contributions

EW and OB designed the study. MH and EP performed the study. All authors contributed to the production of the manuscript.

### Conflict of interest statement

The authors declare that the research was conducted in the absence of any commercial or financial relationships that could be construed as a potential conflict of interest.
